# The Management of Lamb Heterogeneity is a Tool for Farmers’ Marketing Strategies

**DOI:** 10.3390/ani11020551

**Published:** 2021-02-20

**Authors:** Marie-Odile Nozieres-Petit, Charles-Henri Moulin

**Affiliations:** 1 Institut National de Recherche Pour l’agriculture, l’alimentation et l’environnement (Inrae), UMR Systèmes d’Elevage Méditerranéens et Tropicaux (UMR Selmet), 2 place Viala, F-34060 Montpellier, France; 2 L’Institut Agro, Montpellier SupAgro, UMR Systèmes d’Elevage Méditerranéens et Tropicaux (UMR Selmet), 2 place Viala, F-34060 Montpellier, France; moulinch@supagro.fr

**Keywords:** livestock farming system, sheep, sheep meat, marketing, assortment, product, heterogeneity, diversity

## Abstract

**Simple Summary:**

In a value chain, matching supply and demand is needed. Indeed, both vary widely in space and time. The first method of approaching this issue is homogenizing the supply of meat production. It depends on the possibility for farmers to limit the variability in biological processes by improving production conditions. Other marketing strategies are based on the management of animal heterogeneity. In these situations, farmers market a range of products and deal with several market channels.

**Abstract:**

The technical processes used in sheep farming systems are elaborate and difficult to control. The result is a certain heterogeneity in marketed lambs. The aim of this study was to understand how farmers use their practices and modes of marketing to manage and exploit this heterogeneity. We analyzed livestock farming systems in Languedoc-Roussillon (South of France) through eight case studies. We characterize the heterogeneity of lambs during their growth and sale and how the farmers cope with this. Our analysis shows how this heterogeneity, whether intentionally created or merely endured, can be used to invest in different marketing chains. Heterogeneity not only offers adaptable farmers marketing opportunities, but also a method of managing production risks.

## 1. Introduction

The consumption of all food products is broadly regular, frequent, and relatively concentrated in urban areas. Classically, value chains socially function to adjust agricultural production to food consumption, i.e., to match dispersed fluctuating supplies and concentrated regular demand [1]. For sheep meat, performing this function is particularly complex for two reasons. In many countries (e.g., France [2] or Greece [3]), the consumption of lamb meat is occasional and seasonal, punctuated by religious feasts such as Christmas, Easter, or the feast of Eid al-Adha [4]. At the opposite end of the value chain, sheep meat production is subject to high biological variability. Indeed, ovine species are characterized by seasonal reproduction and significant genetic diversity [5]. As a consequence, the production of sheep meat is seasonal and variable [6]. It contains considerable diversity that depends on the breeds used, the location, and the conditions of the territory [7].

Matching supply and demand requires the management of diversity throughout the value chain. The management of product diversity, called product assortment or product mix, is a relatively well-known process in the functioning of value chains and, in particular, as a source of resilience for companies [8,9]. More recently, some studies have analyzed this process for food product value chains [10,11,12], but little or no work asks this question at the level of production systems.

Farmers and sheep meat farmers, like others, are encouraged to invest in the marketing of their products. Two main reasons generate this incentive. The structure of the value chains evolves with, among others, increasing distance between members and their cooperative [13]. Modes of consumption are also changing, with new consumer expectations, including environmental preservation, respect for health, and the search for more local products [14,15]. Local and short supply chains have been developed [16,17].

As a consequence, understanding how breeders contribute to products’ assorted management becomes important and has never been studied. In our work, we explored how sheep meat farmers market their products and especially how they deal with differences between animals. In sheep meat production, irrespective of the different forms of presentation (live animals, carcasses, or wrapped meat), the unit sold is the animal and the transactional object is a batch of animals. Both the management and the sale of lambs (and of ewes) are mainly by batches as they are small animals. In conducting this study, the question we asked was: how and why do sheep meat farmers manage the diversity of young animals with a sales objective?

## 2. Materials and Methods

### 2.1. Study Area

The Languedoc-Roussillon region contains a diversity of natural conditions, all of which are relatively harsh (coastal plains, Mediterranean scrublands, and mountains), which is reflected in the diversity of livestock production systems and the types of animals sold by farmers [18]. The livestock farming systems are mainly present in the hinterland and the dominant regional agricultural productions, vines, arboriculture, and market gardening are located in the plain near the Mediterranean Sea. Among various animal productions, sheep production is predominant, even if the regional herd (around 300,000 ewes, according to our estimates during this work) only represents a small part of the national herd (5,730,000 ewes) [19].

Consumers from Languedoc Roussillon, like those from all around the Mediterranean Sea, appreciate pale pink meat from lighter animals (around 15 kg carcass) than those slaughtered on the national market (around 18 kg carcass). The population in the Languedoc-Roussillon (around 2,600,000 inhabitants, with a positive annual demographic balance, i.e., more people entering than people leaving the territory [20]) eats more sheep meat than in Northern France. This regional consumption is around 156% of the national consumption which was at this date 3.3 kg equivalent carcass weight/inhabitant/year [21]. When consumers buy lamb meat directly to farmers, they are actually buying a half or whole carcasses and therefore get pieces of different quality. The meat is presented already cut and in boxes. Fewer are consumers who buy a whole, uncut, carcass. The cost of slaughter is covered by the farmer, who pays, in accordance with the law, an approved slaughterhouse.

The Languedoc-Roussillon region is located between two big lamb production areas: Provence-Alpes Côte d’Azur and Midi-Pyrénées, which have huge producers’ organizations. Such producers’ groups are promoted by the European legislation with the aim of rebalancing the commercial relations between farmers and the others operators of the downstream sector. All the actors of the downstream sector in Languedoc-Roussillon have developed strategies that are influenced by the proximity of these two dynamic regions.

This situation, i.e., large demand and a wide range of buyers (consumers or operators) located in or near the Languedoc-Roussillon region, offers sheep meat farmers plenty of choice in marketing their animals. We have observed, while conducting this study, that this plenty of choices in sales channels allows breeders to optimize the development of their gross margin. Thus, if they have the resources to finish their animals, the choice of “direct sale” outlets can lead to obtaining producer prices 1.7 times higher than those obtained in longer commercial channels (prices corrected for slaughter and packaging costs but not for extra work).

### 2.2. Sampling and Data Collection

We conducted an exploratory study between autumn 2009 and winter 2010–2011, with 50 surveys of Languedoc-Roussillon sheep meat farmers, to identify their different methods of marketing lambs. 

We define the mode of marketing as a set of product–buyer pairs (PBPs) used by a sheep meat farmer in a campaign. The PBP tool is inspired by the concept of product–market pair used in economic sciences [22]. In our context, a PBP refers to a type of lamb sold associated with a type of buyer. The proportion of each PBP among all the lambs sold in a campaign is specified. If the in situ observation appeared relevant, we include in the description of the mode of marketing the total number of lambs sold in a campaign and the target period for the sales.

Several types of PBPs were observed in the Languedoc-Roussillon region: 19 sheep meat farmers of the 50 used the PBP of light lambs (for fattening) sold to a producers’ organization. The PBP of heavy lambs sold to a producers’ organization was used by 9 sheep meat farmers. Heavy lambs sold to individual consumers was used by 38 farmers. Eighteen sheep meat farmers used the PBP of halal males for individual consumers, mostly around the feast of Eid al-Adha and 13 sold lambs destined for breeding to other farmers. Some sheep meat farmers organized their sales either exclusively or predominantly around one PBP. The others deliberately used several PBPs. We observed that the nature of certain PBPs inevitably requires the creation of others, for example, the use of halal males for individual consumers involves selling the females in another PBP.

Then, we chose 8 of the 50 sheep meat farmers to represent the various marketing modes to monitor them during 2 years. The main features of these eight livestock farming systems are presented in Table 1 and Table 2 in an order of presentation established to promote understanding. Only Farmer 5 used one PBP for marketing. Farmers 1, 2, and 6 used one main PBP. The other four sheep meat farmers chose a sales strategy with several PBPs. We monitored these eight farms over two campaigns (2012 and 2013) by visiting the farmers two or three times a year. In each interview, we collected information on breeding practices combined with the farmer’s points of view [23] on marketing, reproduction, and feeding. Where possible, we collected written data, such as delivery slips, sales receipts, weight tickets, and lambing records. In four cases, we observed sales or sorting for sales. Our analysis is based on these eight case studies, as is common in management science research [24].

### 2.3. Characterization of Heterogeneity through the Analysis of Sheep Meat Farmers’ Practices

From mating to sale, biological variability combined with technical processes results in differences between the lambs marketed in a given year. We refer to these phenotypic differences between animals as heterogeneity. To understand each sheep meat farmer’s strategy in dealing with this heterogeneity, we characterized it into two stages: (1) after the lambing period, at the level of birth cohorts, which we refer to as starting heterogeneity; and (2) at the time of the sale, at the level of the sale sets, which we refer to as marketing heterogeneity. The starting heterogeneity of a cohort (i.e., all the lambs born during the same lambing period as defined by [6]) can be defined as the phenotypic differences between lambs born in the same mating period recorded at the end of the lambing period. As we were unable to measure the quantitative traits of all the lambs at the end of the lambing period, we characterized the starting heterogeneity of a cohort in terms of two reproduction practices: (1) the length of the mating period and (2) the breed of the rams chosen for mating. At the end of the lambing period, the difference in the age of the lambs ranges from one day to several weeks depending on the length of the mating period, resulting in a wide range of live weights. The genetic type of the ram results in differences in both birth weights and growth rates [25,26]. An 8-week-long lambing period and the use of a hardy ram (generally the same breed as the ewes) resulted in medium starting heterogeneity of a cohort. A shorter lambing period reduces it. For a lambing period of four to six weeks, we considered the starting heterogeneity of a cohort to be low, and for a three weeks’ period, very low. Conversely, the longer the lambing period, the higher the starting heterogeneity of a cohort: high for a lambing period ranging from 9 to 11 weeks, and very high for beyond 12 weeks. When rams of two different breeds were used for mating (a hardy ram and a meat ram, for instance), we considered the starting heterogeneity of the cohort to be high. Hence, we defined the overall starting heterogeneity as the phenotypic difference existing among all the lambs born during one campaign and valued it by weighting the starting heterogeneity of all the cohorts in a campaign.

We defined the marketing heterogeneity as the phenotypic difference among lambs sold in the same product–buyer pairs (PBPs). Like for birth cohorts, we were unable to measure marketing heterogeneity. We therefore considered it by cross-analyzing the overall starting heterogeneity, the practices used to raise the lambs up until sale, and the points of view of the farmers regarding their sales batches. To describe the phenotypic differences between lambs, we used the criteria used by the farmers to conduct the different sales transactions, e.g., if the weight and fattening of lambs were used to define the sales price of heavy lambs sold to a producers’ organization. For an occasion like the feast of Eid al-Adha, farmers and consumers include the appearance of the horns in defining the price.

Some practices increased the starting heterogeneity of a cohort and others reduced or at least maintained it. We characterized this in terms of the zootechnical or commercial levers farmers used to manage and orient the heterogeneity between the lambs’ birth up to their sale, to transform the overall starting heterogeneity into overall marketing heterogeneity. The zootechnical levers correspond to the choices made to feed lactating ewes and their lambs and are well-described in the literature [27,28,29]. Thus, Farmers 5 and 6 chose to create batches comprising the mothers of twins. The farmers increased the energy level of the ration for these ewes to enhance milk production and the growth rate of their lambs, which are generally smaller at birth. After weaning, Farmer 8 fed his lambs by putting them in low-potential rangelands to allow them to express different growth rates.

**Figure 1:**
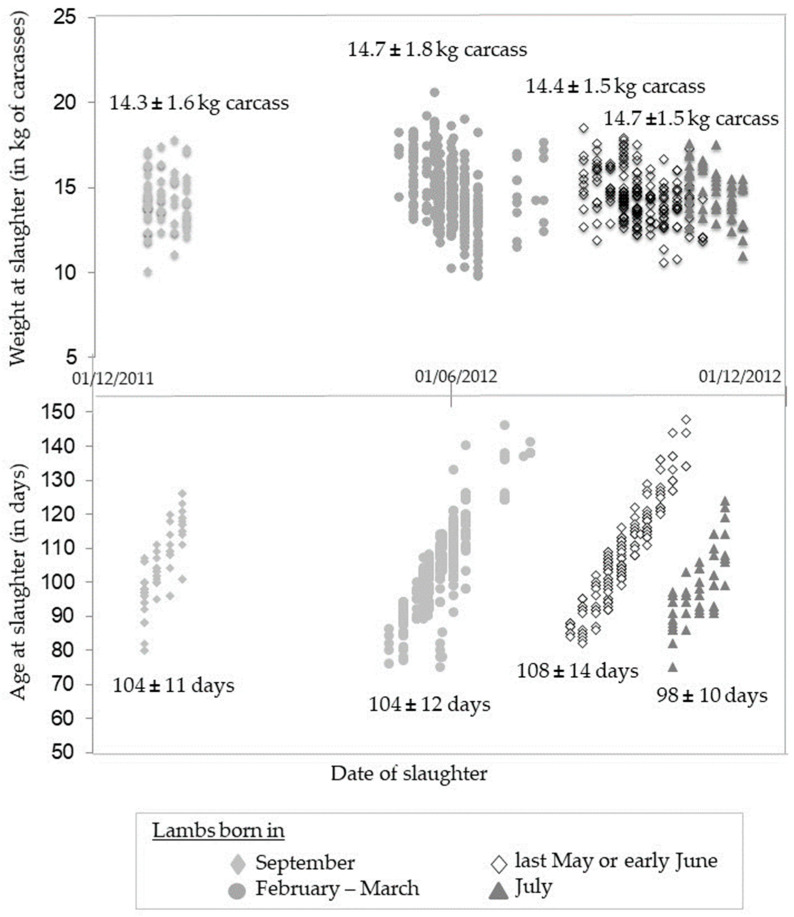
Example of sheep meat Farmer 5 who spreads out the sales to reduce heterogeneity.

We differentiated two commercial levers, of which spreading out sales is one. Using this, farmers sort their lambs to reduce heterogeneity within sales batches according to the criteria they selected as relevant. Farmer 5 used this lever for their four sales periods and created sales batches of lambs of similar weights but of different ages (Figure 1). Thus, for the first sales period, there were four sales batches with 14.3 ± 1.6, 14.7 ± 1.8, 14.4 ± 1.5, and 14.7 ± 1.5 kg carcass weights with age at slaughter of 104 ± 11, 104 ± 12, 108 ± 14, and 98 ± 10 days, respectively. The need for homogenous weights implies an increase in the age of the lambs at the transaction date for the two middle sales batches. This is the only commercial lever available when a farmer uses only one PBP. The other option is the bundling of sales. In extreme cases, this could mean selling all the lambs of a PBP on a given date. This decision may be guided by the type of market sought, such as sales for for Eid al-Adha’s feast, or by other factors, such as the farm schedule (to alternate lambing and sales periods, for example). The number of PBPs is the second commercial lever. By choosing several PBPs, a farmer sorts the lambs among them for sales and thereby manages the marketing heterogeneity for each PBP. Thus, Farmer 6 created three PBPs, each with a low level of marketing heterogeneity. Sometimes the level of marketing heterogeneity differs between the PBPs. For example, Farmer 4 created the PBP of selling heavy lambs to individual consumers by sorting the lambs into the autumn birth cohort, putting the heaviest and the lightest together in one batch, and selling the others in their main PBP of light lambs sold to a producers’ organization with less heterogeneity. The heterogeneity of the batch destined for the collateral PBP was high, as it contained lambs excluded from the main sales batch. The homogeneity of a batch for a PBP with a high demand for standardization can be obtained by sorting animals and accepting heterogeneity in other batches for sale in PBPs with less or no demand for standardization.

Thus, each farmer manages all their birth cohorts from starting heterogeneity to marketing heterogeneity and in a campaign from overall starting heterogeneity to overall marketing heterogeneity. At the end of the process, the marketing heterogeneity of a PBP was considered low when the difference between lambs within one sales batch and between sales batches was as low as possible, as in the case of Farmer 5. Marketing heterogeneity was classified as medium when the difference within and/or between sales batches was medium, and as high when there was a notable difference within and/or between sales batches. Overall marketing heterogeneity was obtained by weighting the marketing heterogeneity of all the PBPs. In the next section, we characterize starting heterogeneity, marketing heterogeneity, overall starting heterogeneity, and overall marketing heterogeneity for the eight farmers, then we compare the different methods of managing lambs and ewes and distinguish different types of strategies.

## 3. Results

### 3.1. Characterization of Starting Heterogeneity

The eight farmers managed a total of 20 birth cohorts. Each farmer managed from one to four cohorts (Table 3). For 15 cohorts, the breeding practices resulted in medium (4 cohorts) or low starting heterogeneity (11 cohorts) due to the short mating period (three to six weeks). For five cohorts, four farmers maximized the starting heterogeneity of a cohort with long periods of mating or crossbreeding. In the end, we distinguished two approaches that led to different levels of overall starting heterogeneity. In the first approach, farmers based all their birth cohorts on the same design. When there was more than one lambing period, the animals were as similar as possible in each cohort, with low overall starting heterogeneity (Farmers 2, 5, and 6), or as different as possible, with high overall starting heterogeneity (Farmer 8). Farmer 7 had only one lambing period, with medium overall starting heterogeneity. In the second approach, farmers used practices that maximized heterogeneity for part of the birth cohorts and reduced it for the remainder (Farmers 1, 3, and 4). The overall starting heterogeneity was medium or low (Farmers 3 and 4, with more than 70% of the lambs with low or medium starting heterogeneity in the cohort), or high (Farmer 1).

### 3.2. Characterization of Marketing Heterogeneity

The eight farmers managed 23 product-buyer pairs (PBPs), ranging from one to five per farmer. For each PBP, we described the difference between lambs at two levels: within and between the sales batches (Table 4), which characterizes marketing heterogeneity. Marketing heterogeneity was low for nine of the 23 PBPs. Different PBPs were included, like selling heavy lambs for a quality label to a producers’ organization (PBP 5-1) or selling heavy lambs to individual consumers (PBP 2-1). However, the marketing heterogeneity of the eight PBPs involving long value chains was generally low (only PBP 2-2, involving a producers’ organization, and 8-1, concerning a wholesaler, were not classified as having low marketing heterogeneity). Marketing heterogeneity was medium for 11 PBPs, 6 of which concerned heavy lambs sold in short value chains. This was also the case when small batches of heavy lambs were sold to a producers’ organization (PBP 2-2) or when batches of light lambs were sold to other farmers (PBP 3-3). Finally, three PBPs were classified as having high marketing heterogeneity, but these only concerned sales to individual consumers or to other farmers.

At the farm level, observing an annual marketing campaign, we differentiated several situations (Table 4). First, Farmers 5 and 6 managed from one to three PBPs with low marketing heterogeneity. Farmers 2, 4, and 7 combined PBPs with low and medium marketing heterogeneity. In these cases, the majority of lambs were sold in a PBP with low marketing heterogeneity, the percentage ranging from 63% and 66% of their lambs sold by Farmers 7 and 4, respectively, to 89% by Farmer 2. Farmer 1 created three PBPs, all with medium marketing heterogeneity. Farmer 3 managed five PBPs with low (35% of all the lambs sold), medium (11% of the lambs sold), and high marketing heterogeneity (52% of the lambs sold). Finally, Farmer 8 managed a balanced proportion of lambs sold (51% versus 30% and 19%) in PBPs with medium and high marketing heterogeneity (Table 4).

### 3.3. Three Strategies to Manage Heterogeneity from Mating to Sales

The first strategy is when farmers create homogenous birth cohorts and try to reduce heterogeneity by the time of sale (Famers 5 and 6) (Table 4). These farmers used both zootechnical and commercial levers (Table 5) to control the heterogeneity of each PBP during the lamb-growing period. The differentiated management of the cohorts in a campaign (Farmers 1, 2, 3, 4, and 7) is the second strategy. In these cases, the famers organized their PBPs to have some with low marketing heterogeneity and others with medium and high marketing heterogeneity. They used levers to increase or decrease heterogeneity. For instance, Farmer 4 had medium overall starting heterogeneity. They organized an initial two-month mating period for purebred animals. In the second, slightly longer mating period, they placed meat and rustic rams in the flock, leading to higher starting heterogeneity. During the growing period of the first birth cohort (81% of the births), the ewes with twins were managed separately from those with one lamb. They received more barley, corn, and second cut hay, while the others received first cut hay. As such, the farmer reduced the variability in milk production and consequently that of the lambs’ growth. The lambs in the first cohort were also fed ad libitum with concentrates to enable each individual to fulfil its growth potential. Farmer 4 then sorted the cohort to build PBP 4-1. The remaining lambs and those of the second birth cohort were fattened using a different feeding strategy to enable direct sales to final consumers each week (PBP 4-2). Finally, the third strategy is described by the creation of heterogeneous birth cohorts and an increase in heterogeneity before the lambs were sold in one or all the PBPs. Farmer 8 organized a six-month mating period plus another of two months and used partial crossbreeding. Ewes grazed outside all year round and were fed hay only on days when the weather was bad. These practices resulted in variable milk production levels and hence variable growth rates. The lambs were managed together after weaning when they were 100 days old. They received no grain, except when the growth rates were so low there was a risk they would not reach the minimum 16 kg carcass weight.

### 3.4. Use of Strategies Depends on Market Chosen by the Farmer

These strategies can differ among the target market(s) chosen by the farmer. Some outlets enable PBPs with medium or high marketing heterogeneity. For example, Farmer 3, sold halal males to individual consumers (PBP 3-1). These lambs were sold as a single batch for the feast of Eid al-Adha, which occurred in autumn during the monitored campaigns. Farmer 3 created a single batch comprising lambs born in the spring lambing period and lambs born in autumn and let them graze in the pasture until Eid al-Adha, providing no complementary feed. Overall, we observed that direct sale is often the channel which best matches high marketing heterogeneity. In this case, demand is highly fragmented and comes from many individual customers, each of whom has different requirements in terms of price or of the characteristics of the lamb they want to buy (weight, fat cover, shoulder height, appearance of horns, etc.) depending on the size of their household, their budget, etc. Other channels require major differences in the animals between sales batches but not within sales batches. As an example, Farmer 1 used PBP 1-1: heavy lambs sold to butchers. Each butcher wants a particular type of lamb (more or less fat cover, different weights, etc.) depending on their customer base. Thus, this PBP 1-1 was sold in small batches, one batch per butcher. Overall, we observed that the channels that require high differences between sales batches but homogeneity within sales batches have few buyers. Finally, some outlets necessitate limiting differences between lambs within and between sales batches. For example, Farmer 5 always sold all their lambs in a single PBP: heavy lambs for a quality label sold to a producers’ organization. Each year, 80% of their production is sold under the quality label Agneau de Lozère, a Protected Geographical Indication. The animals’ characteristics must correspond to the specification. For this reason, Farmer 5 aimed at low overall starting heterogeneity and low overall marketing heterogeneity. Farmers 4 and 7 also tried to limit marketing heterogeneity for their major PBP, light lambs sold to a producers’ organization. This PBP is sold in one sales batch, which is as homogenous as possible. Overall, we observed that low marketing heterogeneity is linked to a demanding market in terms of characteristics. This market requires the same type of lamb all year round, absorbing large and even huge sales batches.

## 4. Discussion

### 4.1. Characterizing Management of Lambs’ Heterogeneity by Analyzing Breeding Practices: Some Advantages

Phenotypic observations or zootechnical measurements are difficult to perform outside experimental conditions and/or for large numbers of farms and animals. Instead, our method is based on the observation of farmers’ practices and the understanding of how they evaluate and manage their animals at different stages. However, no differences between animals is impossible: there is always residual variability. Consequently, the marketing heterogeneity is never null. For a given PBP, as the period of sales advances, the number of animals available decreases and it becomes increasingly difficult to create sales batches with small differences between lambs. In several cases, farmers call their last sales batch “the tail of the cohort”. To analyze an individual farmer’s strategy and to compare the methods used by farmers to manage heterogeneity over the course of the growing process, we must ignore the smallest differences. However, this has an optical effect: the smaller the batch of animals, the bigger the differences between the animals. Assessing the differences between animals thus seems to depend on the size of the batch, which relies on the nature of the PBP. The number of sales batches also depends on the nature of the PBP, among other things.

### 4.2. The Use of Zootechnical and Commercial Levers Requires Sorting Lambs and Ewes

This method is based on a detailed understanding of practices, as defined by [20]. Reproduction practices, e.g., [30] or [6], and feeding practices [31,32] are well-described in the literature. Sorting practices are less well-described. The use of the zootechnical and commercial levers requires and highlights sorting lambs and mother ewes. This type of practice is reported mainly for the mother’s herd [33] or with respect to feedlots [34]. Here, we considered sorting of both lambs and ewes, with the specific objective of marketing. One first type of sorting is called zootechnical levers. Farmers separate lambs or ewe–lamb pairs during pregnancy or growing periods to create batches to manage and, if necessary, adjust complementary feed to energetic needs. The criteria for this type of sorting may be the number of lambs suckled per ewe and the week of birth. For continuing growth and fattening after weaning, the criterion for sorting lambs is generally weight. The second type of sorting occurs before the animals are sold and enables the creation of sales batches. The weight (carcass or live weight) is the main criterion used when the lambs are sold on the meat market. However, the range of weight and the existence of additional criteria depend on the nature of the PBP. For sales based on quality label specifications, non-compliance with certain criteria can lead to some lambs being disqualified. In some cases, sorting occurs early in the growing process, which creates a management batch that prefigures the sales batch. However, in most cases, sorting is performed regularly during the growing period and the sales batches are formed gradually. In rare cases, farmers have only one management batch that includes the whole birth cohort. A few days before the transaction date, using well-defined criteria, the farmers select the best lambs to create one sales batch. They repeat this operation throughout the sales period until the whole cohort is sold. This practice allows them to reduce the marketing heterogeneity for a given PBP. Each farmer applies precise rules when creating the different management and sales batches and know the logical consequences of these rules, but most farmers find it hard to explain them. Sorting requires real expertise. Daily observations are useful to prepare for sorting, possibly tagging of the animals, but without separating them.

### 4.3. Managing Overall Marketing Heterogeneity Is Balanced with Other Sales Objectives

For several PBPs, low overall marketing heterogeneity is advantageous as it helps farmers negotiate a better price for a sales batch. However, to improve their income, farmers try to increase the number of lambs sold per year or provide heavier lambs for sale. Some zootechnical practices or choices for the herd help achieve these two goals. For example, to increase the number of lambs sold, farmers can try to enhance fertility by flushing (Farmers 5 and 6) or by prolonging the mating periods (Farmer 8). They can also improve prolificacy by choosing replacement ewe lambs among the twin births (Farmer 5) or a prolific breed (Farmer 8). However, all these practices lead to higher marketing heterogeneity. In certain cases, for example, Farmer 5, finding the balance between reducing overall marketing heterogeneity for their single PBP and increasing the total number of lambs sold is difficult. Each year, they face a trade-off. In other cases, e.g., Farmer 8, increasing the number of lambs is compatible with the search for a high level of overall marketing heterogeneity. Moreover, heterogeneity is a method of managing production risks.

### 4.4. Heterogeneity Is Managed along the Value Chain

Any heterogeneity remaining after the farmer has sold the animals is managed by downstream operators. This requires real expertise regarding the customer to whom each animal will be sold based on its characteristics. Sorting is performed by each supply chain operator, first as live animals, then as carcasses, and finally as wrapped meat. Although this process is well-recognized and documented in other sectors of the economy [8,9], it is less well-documented in the agro-food sector [10,11,12]. It is nevertheless essential to match supply and demand. Sorting is also the basis for market segmentation to structure an offer with some quality products [35,36]. What creates quality, i.e., the characteristics of quality products, is defined and negotiated between the different operators of the value chain, serving to define the main principles used to manage heterogeneity. In direct sales, it is limited by the small number of operators (only two: the producer and the consumer). The need for specific expertise for sorting wrapped meat is not always recognized by the farmer, who assumes the role of the retailer. This creates a risk of animals remaining unsold. In the other types of sales, operators all along the value chain are specialized managing heterogeneity. Small-scale traders of live animals have thus developed considerable expertise in managing the heterogeneity of animals and basically build their business with this aspect in mind.

### 4.5. Does Management of Heterogeneity Exist in Other Cases?

The heterogeneity of animals is an intrinsic characteristic of living beings, and is useful for structuring and managing the diversity of animals [37]. However, encouraging diversity to promote the transition to agroecology is widely accepted [38,39] and is especially interesting in relation to building the resilience of systems [40,41]. On the breeding system scale, works have essentially been structured on the analysis of the diversity of breeding females and, in particular, on genetic and functional diversity and its capacity to build and maintain zootechnic performance [42]. Some studies have attempted to understand the management of the diversity of breeding females [43,44,45,46].

Little or no research has been carried out to analyze how farmers deal with the biological heterogeneity of their animals for marketing. This type of analysis is, however, carried out in the downstream sectors or in other sectors of the economy (see above). The analysis we are proposing here could be developed, in sheep meat productions and other situations, in order to support farmers in their marketing choices and the consequences that this may have on their technical practices. It would highlight the farmers’ know-how of sorting and assortment, which they have like the other operators in the value chain, and which they could promote. Finally, it would be beneficial to identify situations where the traceability of products from birth can be complex. Nevertheless, led to this scale of livestock farming systems, this type of analysis mobilizes knowledge already produced elsewhere, in beef or sheep production, like the analysis of breeding practices and their relationship with performance, but also the analysis of ranges of products sold by farmers. On this last point, these works [47,48] show in particular the interest of a diversity of products in the search for resilience and flexibility of production systems.

In our study, we observed several levels of overall starting heterogeneity molded with different management strategies to produce several levels of overall marketing heterogeneity. This may be due to some characteristics of the Languedoc-Roussillon region. First of all, even if the environments are diverse, they can be globally qualified as harsh environments. This fact is probably one of the reasons for the wide range of overall starting heterogeneity observed. Secondly, the proximity of, on the one hand, a large consumption area, and, on the other hand, two regions with developed downstream sectors, probably encourage different levels of overall marketing heterogeneity by multiplying potential outlets. This may differ in other regions where the relationship between supply and demand is not the same and/or where the geophysical environments are more favorable. We hypothesize, in particular, that in such regions, management to reduce overall marketing heterogeneity is common. This joins some conclusions of works like [49], but this hypothesis needs to be confirmed.

The choice of sheep meat production also influences our results. In cattle production, the size of the animals is bigger and the sales batches are small, and may even be limited to a single individual (a practice that is rare in sheep production). The heterogeneity within or between batches of cattle is theoretically less of a management issue. Market segmentation is clearer in the case of cattle. In the case of live animals, the distinction between animal products provided by all the actors of the supply chain is more stable. This ensures that building different PBPs is an effective method of managing heterogeneity.

## 5. Conclusions

In this study: we provided a new view on the transactions between livestock farmers and those who purchase their products, and the management of livestock to prepare and succeed in these transactions. To this end, we assessed the heterogeneity of animals at different levels, ranging from lambing cohorts to sales batches, and by practices, ranging from mating to sales. The management of heterogeneity is an asset for building the economic of the livestock production system, as it can be used to negotiate a better price in the transaction. To analyze and support the management of the diversity of young animals is not only useful for structuring the modes of marketing, but can also provide a tool for developing the resilience of systems and hence their entry into the agro-ecological transition.

## Figures and Tables

**Table 1:** Characteristics of the eight sheep meat farmers monitored in this study: flock and zootechnical choices.

Farmer	Breed of Ewes	Number of Ewes	Prolificacy	Annual Productivity	Number of Lambs Sold per Mated Ewe
5	Hardy breed ^1^	1250	Medium	0.94	0.74
6	Hardy breed	455	Medium	1.26	1.11
2	Hardy breed	500	Low	0.94	0.76
4	Rare hardy breed ^2^	300	Low	1.04	0.90
7	Rare hardy breed ^2^	220	Low	0.82	0.62
1	Hardy breed	600	Low	0.77	0.60
3	Hardy breed	900	Medium	1.14	0.92
8	Hardy and prolific breed ^3^	380	High	1.58	1.42

^1^ Blanche du Massif Central or Merino; ^2^ Caussenarde des Garrigues or Rouge du Roussillon; ^3^ Romane. Low = approximately 1 lamb/ewe; Medium = 1.3 to 1.5 lambs/ewe; High = more than 2 lambs/ewe.

**Table 2:** Characteristics of the mode of marketing used by the eight sheep meat farmers monitored in this study: for each famer, a set of product–buyer pairs (PBPs).

Farmer	No. of PBPs	Proportion of Sales (%)	Name of the PBP
5	5-1	100	Heavy lambs for a quality label–Producers’ organization
6	6-1	77	Heavy lambs–Cattle dealers Breeding males–Producers’ organization Breeding females–Farmers
	6-2	16	
	6-3	7	
2	2-1	89	Heavy lambs–Individual consumers Heavy lambs–Producers’ organization Heavy lambs–Local retailers
	2-2	6	
	2-3	5	
4	4-1	63	Light lambs–Producers’ organization Heavy lambs–Individual consumers
	4-2	37	
7	7-1	66	Light lambs–Producers’ organization Heavy lambs–Individual consumers Breeding males–Farmers
	7-2	31	
	7-3	3	
1	1-1	77	Heavy lambs–Butchers Heavy lambs–Individual consumers Halal Males– Individual consumers
	1-2	17	
	1-3	6	
3	3-1	52	Halal Males– Individual consumers Breeding Females–Farmers Heavy females lambs–Individual consumers Light lambs–Farmers Breeding Males–Producers’ organization
	3-2	35	
	3-3	7	
	3-4	4	
	3-5	2	
8	8-1	51	Organic heavy lambs–Wholesalers Heavy lambs–Individual consumers Light lambs–Farmers
	8-2	31	
	8-3	19	

**Table 3:** Overall starting heterogeneity for the eight sheep meat farmers monitored.

F ^1^	Cohort	Lambing Period		Choice of Rams	Starting Heterogeneity of the Cohort	Proportion of Total Births (%)	OSH ^2^
		Length (Months)	Period				
5	1	1	October	Pure Breeding	Low	24	Low
	2	1	February-March	Pure Breeding	Low	38	
	3	0.75	May-June	Pure Breeding	Very low	29	
	4	1	July	Pure Breeding	Low	10	
6	1	2	Sept-October	Pure Breeding	Medium	30	Low
	2	1	December	Pure Breeding	Low	33	
	3	1.5	April	Pure Breeding	Low	37	
2	1	2	February-March	Pure Breeding	Medium	43	Low
	2	1	May	Pure Breeding	Low	7	
	3	1	October	Pure Breeding	Low	49	
3	1	0.75	October	Pure Breeding	Very Low	11	Low
	2	1.5	Nov-December	Pure Breeding	Low	61	
	3	3.5	February-May	Pure Breeding	Very High	28	
4	1	2	Sept-Octobe	Pure Breeding	Medium	81	Medium
	2	2.5	April-June	Partial Crossbreeding	High	19	
7	1	2	September	Pure Breeding	Medium	100	Medium
1	1	4	February-May	Pure Breeding	Very High	69	High
	2	1.5	Oct-November	Pure Breeding	Low	31	
8	1	6	Sept-February	Partial Crossbreeding	Very High	40	High
	2	2	March-April	Partial Crossbreeding	High	60	

^1^ F = farmer; ^2^ Overall Starting Heterogeneity.

**Table 4:** Overall marketing heterogeneity for eight sheep meat farmers monitored.

F ^1^	No. PBP ^2^	Proportion of Sales (%)	Degree of Difference between Lambs		No. Sales Batches	Size of a Sales Batch	MH ^3^	OMH ^4^
			Within Sales Batches	Between Sales Batches				
5	5-1	100	Low	Low	Many	Large	Low	Low
6	6-2	16	Low	Low	A few	Small	Low	Low
	6-1	77	Low	Low	Many	Large	Low	
	6-3	7	Low	Only one batch	One	Large	Low	
2	2-1	89	Low	Low	Many	Large	Low	Medium
	2-2	6	Medium	Medium	A few	Small	Medium	
	2-3	5	Medium	Medium	A few	Small	Medium	
4	4-1	63	Low	Only one batch	One	Large	Low	Medium
	4-2	37	Medium	Medium	Many	Small	Medium	
7	7-1	66	Low	Only one batch	One	Large	Low	Medium
	7-2	31	Medium	Medium	Many	Small	Medium	
	7-3	3	One lamb	Medium	A few	Small	Medium	
1	1-1	77	Low	Medium	Many	Large/small	Medium	Medium
	1-3	6	Medium	Only one batch	One	Small	Medium	
	1-2	17	Medium	Medium	A few	Small	Medium	
3	3-1	52	High	Only one batch	One	Large	High	High
	3-5	2	One lamb	Low	A few	Small	Low	
	3-2	35	Low	Low	A few	Large	Low	
	3-3	7	Medium	Medium	A few	Large/small	Medium	
	3-4	4	Medium	Medium	A few	Large	Medium	
8	8-1	51	Medium	Medium	Many	Large	Medium	High
	8-2	30	High	High	Many	Large/small	High	
	8-3	19	High	High	A few	Large	High	

^1^ F = Farmer; ^2^ PBP = product–buyer pair; ^3^ MH = marketing heterogeneity; ^4^ OMH = overall marketing heterogeneity; Small = less than 10 lambs; Large = more than 10 lambs.

**Table 5:** Levers used to manage heterogeneity by the eight sheep meet farmers monitored.

F ^1^	No. of Cohorts	OSH ^2^	PBP No.	Levers to Reduce Heterogeneity		Levers to Increase Heterogeneity	
				Zootechnical	Commercial	Zootechnical	Commercial
5	4	Low	5-1	Feeding ewes	Spreading sales		
6	3	Low	6-2	Feeding ewes and lambs	Sorting lambs and spreading sales		
			6-1	Feeding ewes and lambs	Sorting lambs and spreading sales		
			6-3	Feeding ewes and lambs	Sorting lambs and spreading sales		
2	3	Low	2-1	Feeding ewes	Sorting		Bundling sales
			2-2				
			2-3				
4	2	Medium	4-1	Feeding ewes and lambs	Sorting		
			4-2		Spreading sales	Feeding lambs	
7	1	Medium	7-1	Feeding ewes and lambs			
			7-2				
			7-3				
1	2	High	1-1	Feeding ewes and lambs	Sorting		
			1-3				
			1-2				Bundling sales
3	3	Low	3-1		Sorting	Feeding lambs	Bundling sales
			3-5				
			3-2				
			3-3				
			3-4				
8	2	High	8-1				
			8-2			Feeding lambs	
			8-3			Feeding lambs	

^1^ F = Farmer; ^2^ Overall Starting Heterogeneity.

## Data Availability

Data sharing not applicable.
